# Development of an immune-related gene prognostic risk model and identification of an immune infiltration signature in the tumor microenvironment of colon cancer

**DOI:** 10.1186/s12876-023-02679-6

**Published:** 2023-03-08

**Authors:** Mengdi Hao, Huimin Li, Meng Yi, Yubing Zhu, Kun Wang, Yin Liu, Xiaoqing Liang, Lei Ding

**Affiliations:** 1 Department of Oncology, Beijing Shijitan Hospital, Capital Medical University, No. 10, Tieyi Road, Haidian District, Beijing, 100038 China; 2 Department of Oncology, Ninth School of Clinical Medicine, Peking University, Beijing, 100038 China

**Keywords:** Immune gene, Prognosis, Risk model, Colon cancer

## Abstract

**Background:**

Colon cancer is a common and highly malignant tumor. Its incidence is increasing rapidly with poor prognosis. At present, immunotherapy is a rapidly developing treatment for colon cancer. The aim of this study was to construct a prognostic risk model based on immune genes for early diagnosis and accurate prognostic prediction of colon cancer.

**Methods:**

Transcriptome data and clinical data were downloaded from the cancer Genome Atlas database. Immunity genes were obtained from ImmPort database. The differentially expressed transcription factors (TFs) were obtained from Cistrome database. Differentially expressed (DE) immune genes were identified in 473 cases of colon cancer and 41 cases of normal adjacent tissues. An immune-related prognostic model of colon cancer was established and its clinical applicability was verified. Among 318 tumor-related transcription factors, differentially expressed transcription factors were finally obtained, and a regulatory network was constructed according to the up-down regulatory relationship.

**Results:**

A total of 477 DE immune genes (180 up-regulated and 297 down-regulated) were detected. We developed and validated twelve immune gene models for colon cancer, including SLC10A2, FABP4, FGF2, CCL28, IGKV1-6, IGLV6-57, ESM1, UCN, UTS2, VIP, IL1RL2, NGFR. The model was proved to be an independent prognostic variable with good prognostic ability. A total of 68 DE TFs (40 up-regulated and 23 down-regulated) were obtained. The regulation network between TF and immune genes was plotted by using TF as source node and immune genes as target node. In addition, Macrophage, Myeloid Dendritic cell and CD4^+^ T cell increased with the increase of risk score.

**Conclusion:**

We developed and validated twelve immune gene models for colon cancer, including SLC10A2, FABP4, FGF2, CCL28, IGKV1-6, IGLV6-57, ESM1, UCN, UTS2, VIP, IL1RL2, NGFR. This model can be used as a tool variable to predict the prognosis of colon cancer.

## Background

Colon cancer is the leading human cancer, accounting for about one in 10 of all cancer cases, and thus ranks third in incidence and second in mortality. As one of the most common malignant solid tumors worldwide, the incidence of colon cancer has been increased year by year [1, 2]. A combination of genetic, dietary, immune, and intestinal microenvironment factors would contribute to tumor development and progression of colon cancer. Neoadjuvant and adjuvant chemotherapy have been widely applied in the treatment of colon cancer. With the emergence of new modalities of molecular targeted therapy and immunotherapy, the survival rate of colon cancer has been improved, however, the incidence of colon cancer keeps increasing rapidly and its 5 year survival rate remains relatively low [3, 4]. Therefore, for colon cancer patients, the discovery of new biomarkers to effectively assess survival risk and prognosis would be urgently needed.

Colonic malignancies correlate with a disrupted or dysregulated immune system. Cytokines secreted by tumor cells can inhibit the secretion of Th1-type cytokines and promote the production of Th2-type cytokines, leading to immune escape. According to the NCCN guidelines for colon cancer (Version 2.2021) [5], immunotherapy has become an essential part of the standard regimen for colon cancer. However, inadequate immune response has caused inconsistent efficacy of immunotherapy. For example, PD-1/PD-L1 or CTLA4 inhibitors have greater efficacy in colon cancer patients with microsatellite instability, while for patients with microsatellite stability, immunotherapy is ineffective due to low tumor immunogenicity [6, 7].

Therefore, how to develop immunogenicity-related prognostic models for colon cancer and to identify features of immune infiltration in tumor microenvironment (TME) of colon cancer will be critical to optimize immunotherapy. In this study, based on gene expression profile in The Cancer Genome Atlas (TCGA), Immunology, The Immunology Database and Analysis Portal (ImmPort), and Tumor Immune Estimation Resource (TIMER) databases, we systematically analyzed the immune gene expression profile, and constructed a risk model to predict clinical outcome of colon cancer. The model facilitates individualized risk assessment of patients. We identified features of immune infiltration in the TME. Notably, immune dysregulation contributed to a suppressive and pathologic TME, providing directions for the discovery of new molecular markers and novel therapeutic targets for immunotherapy of colon cancer.

## Methods

### Databases and statistical analysis

Transcriptomic and clinical data were downloaded from the TCGA (https://tcga-data.nci.nih.gov) database [8]. Immunogenic data were downloaded from the ImmPort (https://www.immport.org/home) database [9, 10]. Immune infiltration data were downloaded from the TIMER (http://cistrome.org/TIMER) database [11]. The differentially expressed transcription factors (TFs) were downloaded from the Cistrome (http://cistrome.org/) database [12]. Immune-cell content was downloaded from timer.cistrome.org. The statistical analysis in this study was generated by R-4.1.0.

### Identification of differentially expressed (DE) genes

The limma R package was installed and immunogenic difference analysis was performed with Wilcoxon signed rank test. The threshold value of FDR and logFC was set-up at 0.05 and 1, respectively. The Pheatmap R package was used to describe differentially expressed genes (DEGs). The genes with a FDR < 0.05 and logFC > 1 were marked in red whereas those with a FDR < 0.05 and logFC < -1 were marked in green, to create a volcano map.

### Identification of survival-associated DE immune genes

The DE immune genes were screened from DE genes and immune genes. According to the American Joint Committee on Cancer (AJCC), patients with survival time less than 90 days or unknown were excluded. Using the SURVIVAL R package, single-factor Cox regression with a filtering criterion set at a *p* = 0.02 was used to obtain DE immune genes associated with prognosis and to draw a forest plot.

### Bioinformatics

Totally, 318 transcription factors (TFs) obtained from the Cistrome database were combined with DE gene set. False Discovery Rate (FDR) and log fold change (log FC) was setup at 0.05 and 1, respectively, to obtain DE TFs, to plot heat map and volcano map.

The coefficient filtering criterion was selected as 0.4 (0.3–0.8), *p* < 0.001. After excluding normal samples, TF was correlated with DE immune genes to screen out up-regulated (> 0.4) and down-regulated (< − 0.4) genes, respectively. With HR = 1 as the cut-off point, the screened genes were divided into high-risk group (HR > 1) and low-risk group (HR < 1). Immune genes were used as target points and TFs as resource nodes to obtain regulatory network of DE immune genes associated with TFs and prognosis using JVA and cytoscape software [13].

### Construction and validation of the prognostic risk model

Using the prognosis-related immune genes, multi-factor analysis was performed and optimized based on significantly associated single factors to determine the best fit model. The expression, coefficient and Hazard ratio (HR) values involved in the model were output. The risk score of a sample was calculated based on the combination of Cox coefficient and gene expression. The risk score was equal to the sum of the product of expression and coefficient of each gene involved in the model construction. To validate its accuracy, survival R package was used to define high risk/low risk genes with HR values. The median was defined as the threshold value to divide genes into high- vs. low-risk group, with high-risk indicating a lower survival rate in colon cancer patients. Survival R package and Survminer R package were used for subsequent analysis and plotted graphically. Furthermore, ROC was plotted using the survROC R package to obtain 1, 3, and 5 year overall survival (OS) to assess the accuracy of a candidate prognostic model. The area under the ROC (AUC) > 0.7 indicated a high accuracy of a model. Patients in different groups were ranked from the smallest to the largest based on risk scores, so that risk score distribution plots and survival status scatter plots could be drawn. Immune genes involved in the model construction were extracted and heat maps were drawn for high- and low-risk groups. Together with ROC curves, these genes were used to evaluate the model. Moreover, we used the TCGA database to verify the prognostic risk model. In addition to the ROC curve, risk score distribution plots and survival status scatter plots were also used to evaluate the accuracy of the model.

### Independent prognostic value of this new model

To explore if this new model could independently assess prognosis, survival analysis was performed and forest plots drawn for colon cancer using univariate and multifactorial cox regression models adjusted with demographic and clinical characteristics (including age, sex, grade, T stage, N stage, and M stage). The parameter with a *p* < 0.05 was identified as an independent prognostic variable.

### Clinical application of this new model

The clinical applicability was evaluated. Correlation analysis was performed between constructed model and demographic/clinical traits, including age (≤ 65 years, > 65 years), gender (male, female), stage (stage I&II and stage III&IV), T-stage (T1-2, T3-4), N-stage (N0, N+), and M-stage (M0, M1). Parameters were divided into two groups according to demographic/clinical traits, while the differences between the two groups was considered statistically significant when a *p* < 0.05 by an independent t-test.

### Identification of an immune infiltration signature

To assess if this new model could reflect the status of the TME of colon cancer, immune-cell contents in different databases were downloaded. After excluding normal samples, different immune cell contents in tumor patients were obtained, which were divided into high- vs. low-risk groups according to the constructed risk model. The pheatmap R package was used to draw heat maps. Patients' immune cell contents were analyzed for correlation with risk scores. *p* < 0.05 was considered as a good correlation, with cor > 0 being a positive correlation whereas cor ≤ 0 being a negative correlation.

The limma R package, reshape2 R package, ggplot2 R package, ggpubr R package were installed. Immune checkpoint genes significantly differentially expressed were extracted using Wilcoxon signed rank tests. The differences of immune checkpoint genes between high- and low-risk groups in the constructed model were analyzed and box line plots drawn to visualize the differences.

## Results

### Basic information

**Fig. 1:**
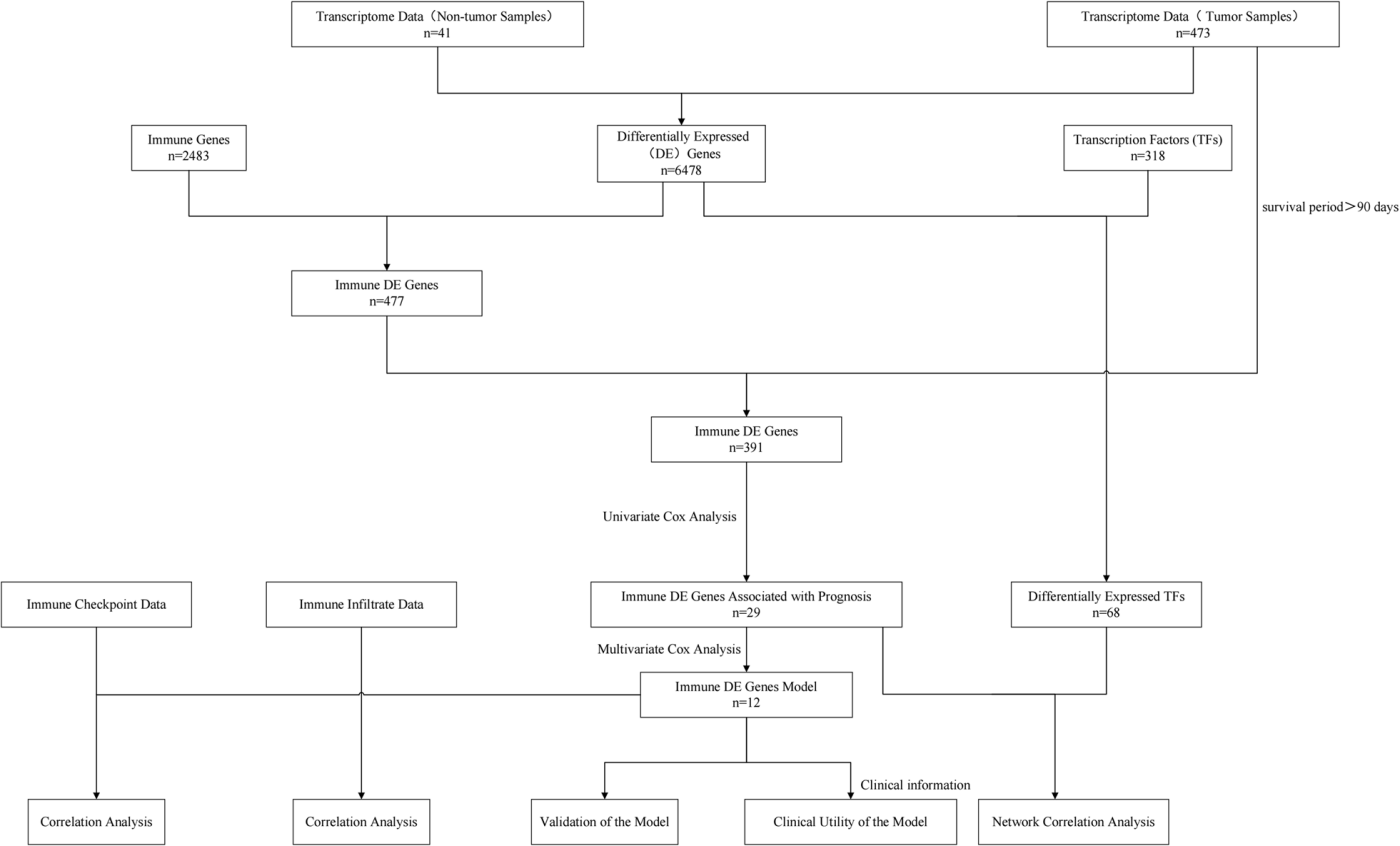
Diagram of the study

**Fig. 2:**
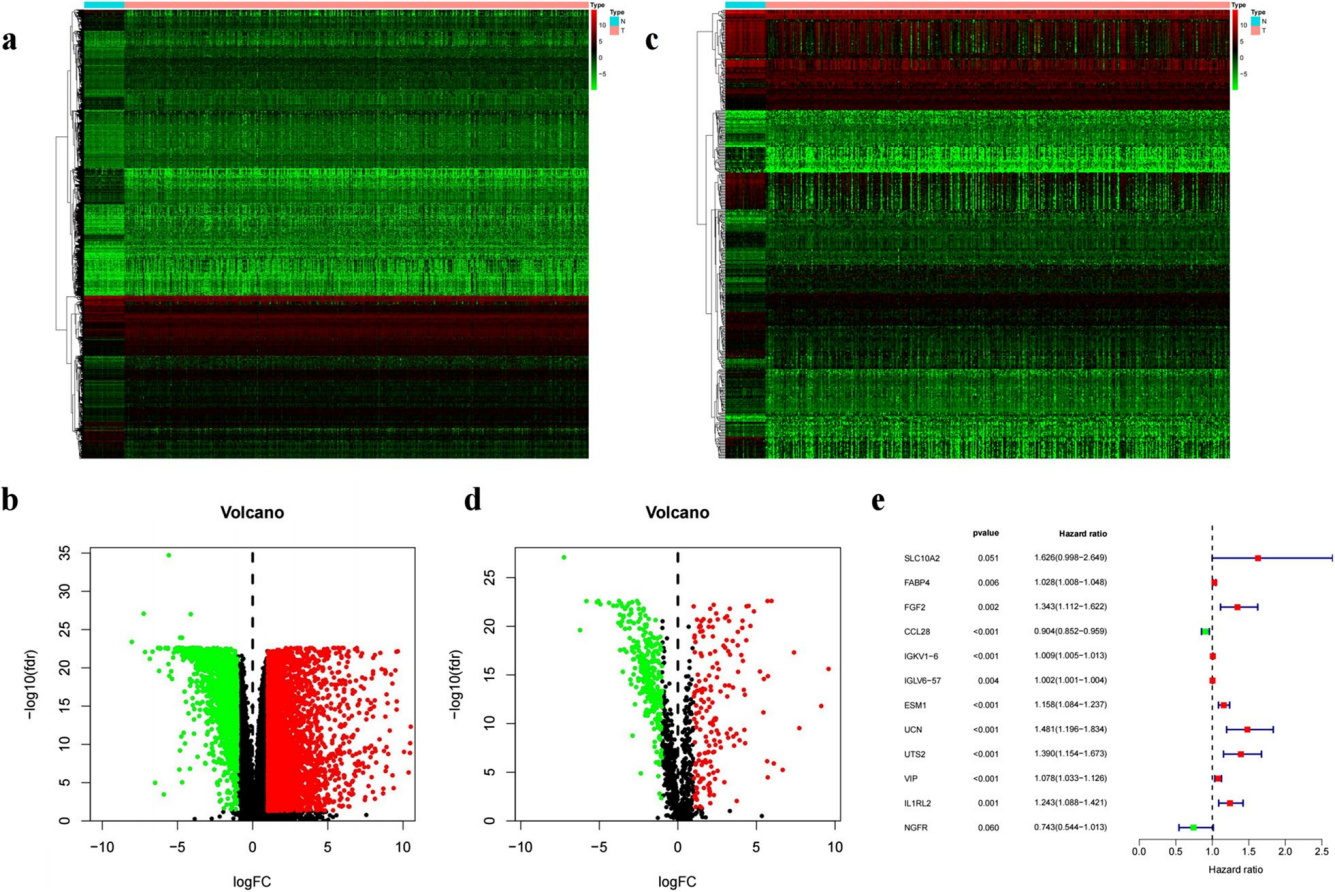
Identification of differentially expressed (DE) immune genes. **a** Heat map of the DE genes. **b** Volcano plot of the DE genes. **c** Heat map of the DE immune genes. **d** Volcano plot of the DE immune genes. **e** Univariate Cox analysis

The workflow was described in Fig. 1. Data of 514 samples were downloaded from TCGA database, including 473 tumors and 41 normal samples. Our study was conducted on this basis. After screening, 6478 differential genes were obtained, including 4562 up-regulated and 1916 down-regulated (Fig. 2a and b). The immune genes were downloaded from immport database, and 2483 immune genes were obtained, and then intersected with derived differential genes. 477 differentially expressed immune genes were identified (Fig. 2c and d), with 180 up-regulated and 297 down-regulated. Survival time > 90 days was considered valuable for prognostic analysis. DE immune genes of 391 samples with survival time > 90 days were included. Thus, 29 differential immune genes associated with prognosis after single-factor Cox analysis were obtained (Fig. 2e).

### Bioinformatics analyses

**Fig. 3:**
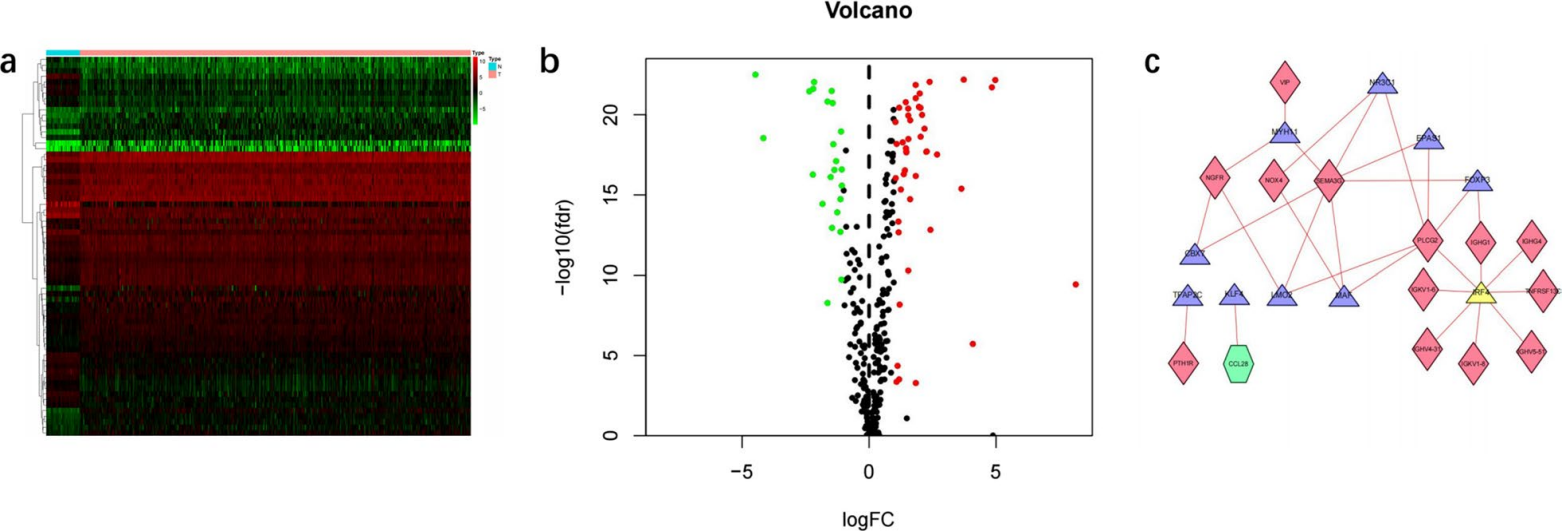
Identification of differentially expressed (DE) transcription factors (TFs). **a** Heat map of the DE TFs. **b** Volcano plot of the DE TFs. **c** A regulatory network of TFs and immune genes

Three hundred and eighteen tumor-related TFs were obtained from Cistrome database, with screening criteria of FDR = 0.05 and logFC = 1. Finally, 68 DE TFs were obtained, including 40 up-regulated and 23 down-regulated. The volcano plot and heat map of DE TFs were drawn (Fig. 3a and b).

The expression levels of TFs were tested for correlation with immune genes, and thus defined as high-risk vs. low-risk based on HR values. Finally, 68 differential TFs were obtained, including 26 high-risk genes and 2 low-risk genes. The TF was used as a source node, an immune gene as a target node, so that a regulatory network of TFs and immune genes could be drawn (Fig. 3c). A total of 29 upregulated genes satisfied the screening criteria.

### Construction and validation of an immune-gene expression model in colon cancer

**Fig. 4:**
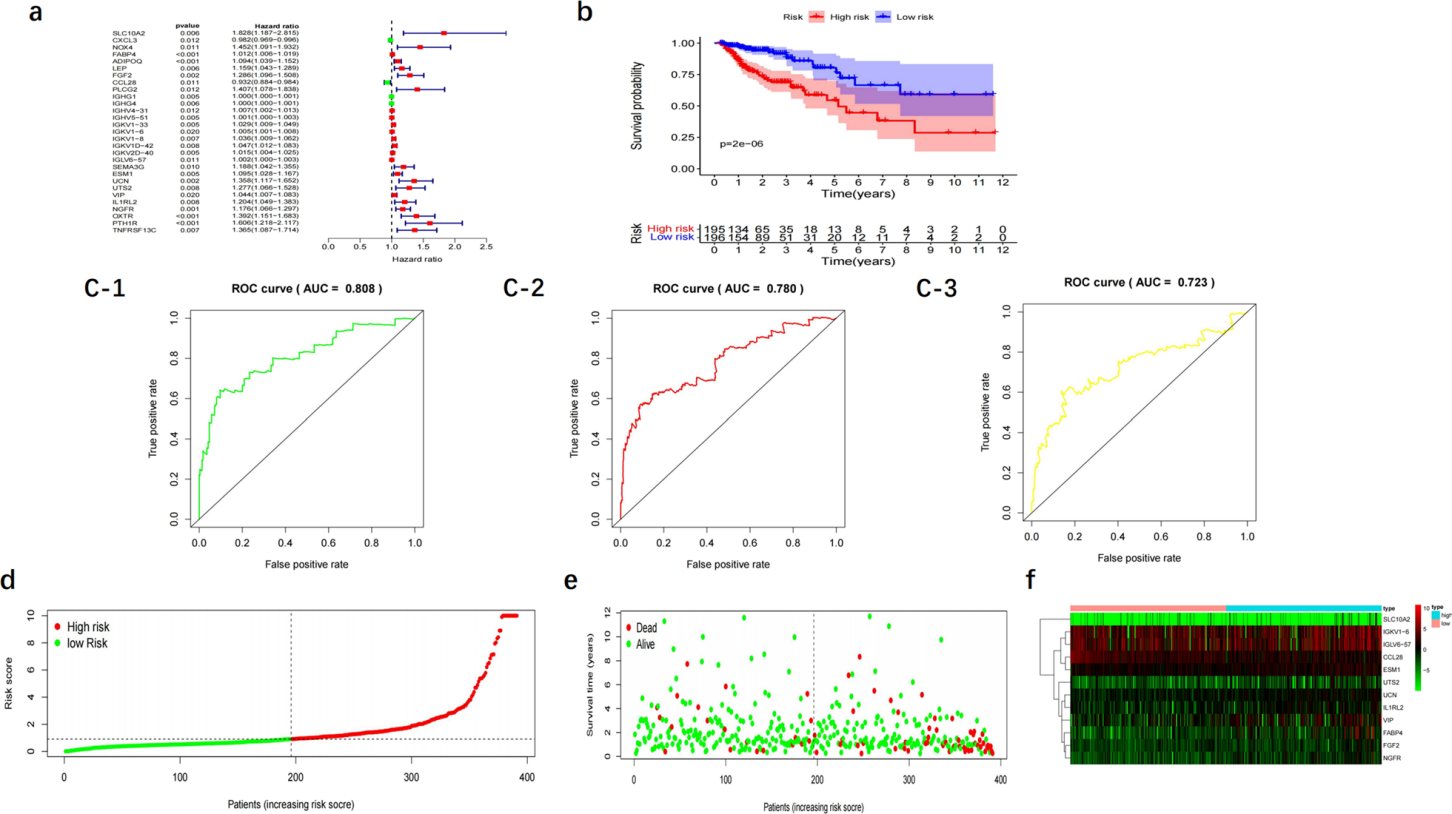
Construction of the prognostic risk model. **a** Multivariate Cox analysis. **b** Overall survival (OS) in the training cohort. **c** Time-dependent receiver operating characteristic (ROC) curve analysis (**c-1**:ROC 1 year, **c-2**:ROC 3 year, **c-3**:ROC 5 year) in the training cohort. **d** Risk score distribution plot in the training cohort. **e** Survival status scatter plots in the training cohort. **f** Heatmap of risk genes

Multi-factor Cox regression was performed on 29 prognosis-related immune genes obtained from single-factor Cox regression analysis. 12 immune genes were screened into a new model (Fig. 4a), including SLC10A2 (solute carrier family 10 (sodium/bile acid co-transport protein family), FABP4 (fatty acid binding protein 4) FGF2 (fibroblast growth factor 2), CCL28: chemokine (C-C motif) ligand 28, IGKV1-6 (immunoglobulin Kappa variable 1-6), IGLV6-57 (immunoglobulin Lambda variable 6-57), ESM1 (endothelial cell-specific molecule 1), UCN (urocortin), UTS2 (Urotensin II), VIP (vasoactive intestinal peptide), IL1RL2 (interleukin 1 receptor-like 2), and NGFR (nerve growth factor receptor). The risk score was calculated as follows: risk score = 0.4861* SLC10A2 expression value + 0.027458* FABP4 expression value + 0.294596* FGF2 expression value − 0.10134* CCL28 expression value + 0.009209* IGKV1-6 expression value + 0.002103* IGLV6-57 expression value + 0.146603* ESM1 expression value + 0.3926* UCN expression value + 0.329081* UTS2 expression value + 0.075367* VIP expression value + 0.21784* IL1RL2 expression value − 0.29771* NGFR expression value.

**Fig. 5:**
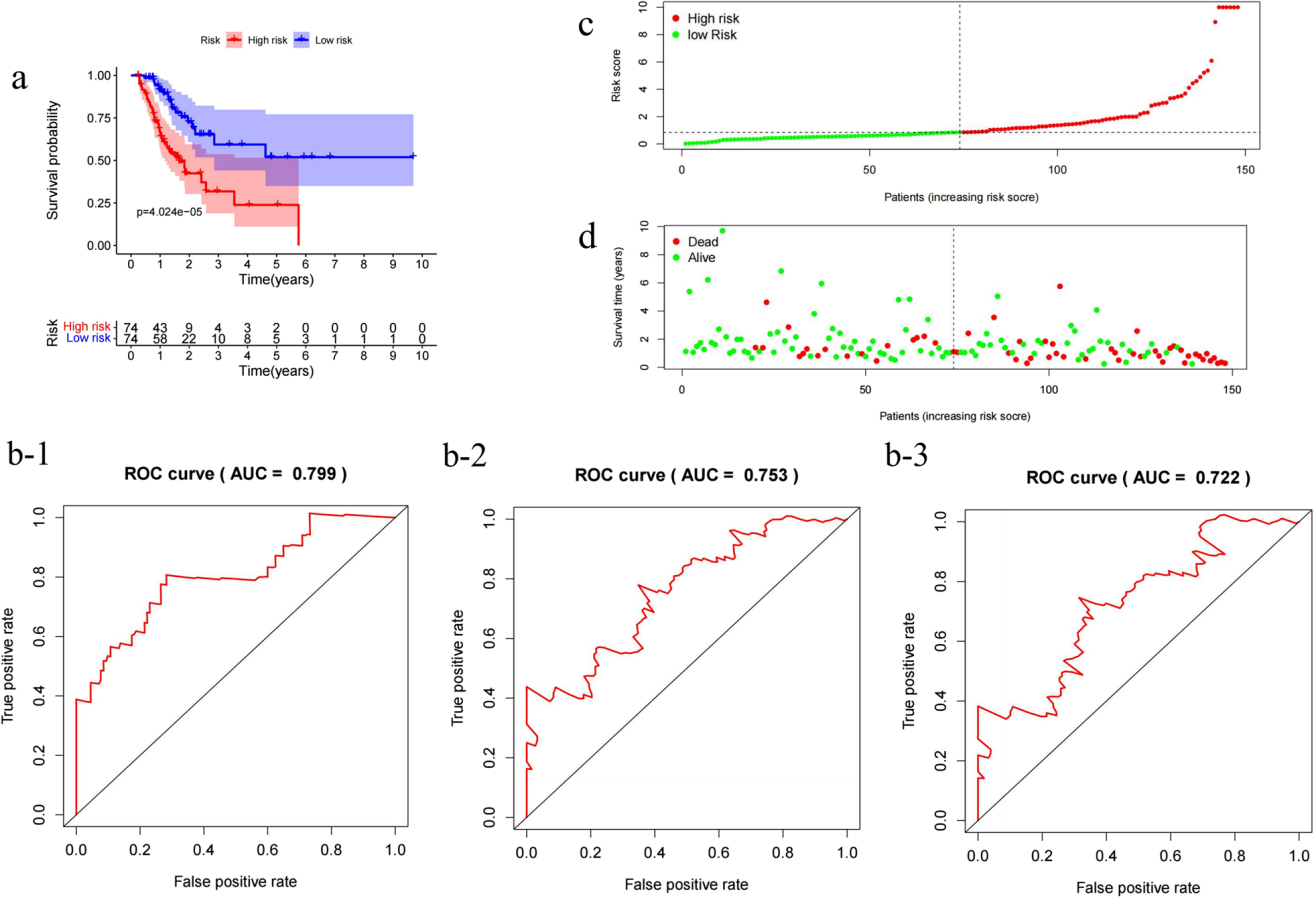
Validation of the prognostic risk model. **a** Overall survival (OS) in the validation cohort. **b** Time-dependent receiver operating characteristic (ROC) curve analysis (**b-1**:ROC 1 year, **b-2**:ROC 3 year, **b-3**:ROC 5 year) in the validation cohort. **c** Risk score distribution plot in the validation cohort. **d** Survival status scatter plots in the validation cohort

Then, all samples were divided into high-risk and low-risk groups based on median risk score values. 195 colon cancer patients were classified into the high-risk group whereas 195 into the low-risk group. A significant difference in the survival rate was observed between the two groups (*p* < 0.05) (Fig. 4b), with a 5 year survival rate of 49.5% (95% CI 0.363–0.676) in high-risk group whereas 76.3% (95% CI 0.647–0.900) in low-risk group. The AUC value of immunogenetic prognostic model at 1, 3 and 5 years OS were 0.808, 0.780, and 0.723, respectively (Fig. 4c-1–c-3). (Fig. 4d–f represents the distribution of risk scores, survival status and risk gene expression heatmap of high-and low-risk groups). To verify its accuracy, we drew the ROC curve of the validation cohort (Fig. 5). The AUC value of validation cohort at 1, 3 and 5 years OS were 0.799, 0.753, and 0.722 (AUC > 0.7) respectively, indicating this model might accurately predict the prognosis of colon cancer patients. The risk score distribution plot and survival status plot was shown in Fig. 5.

### Independent prognostic value and clinical utility of this risk model

**Fig. 6:**
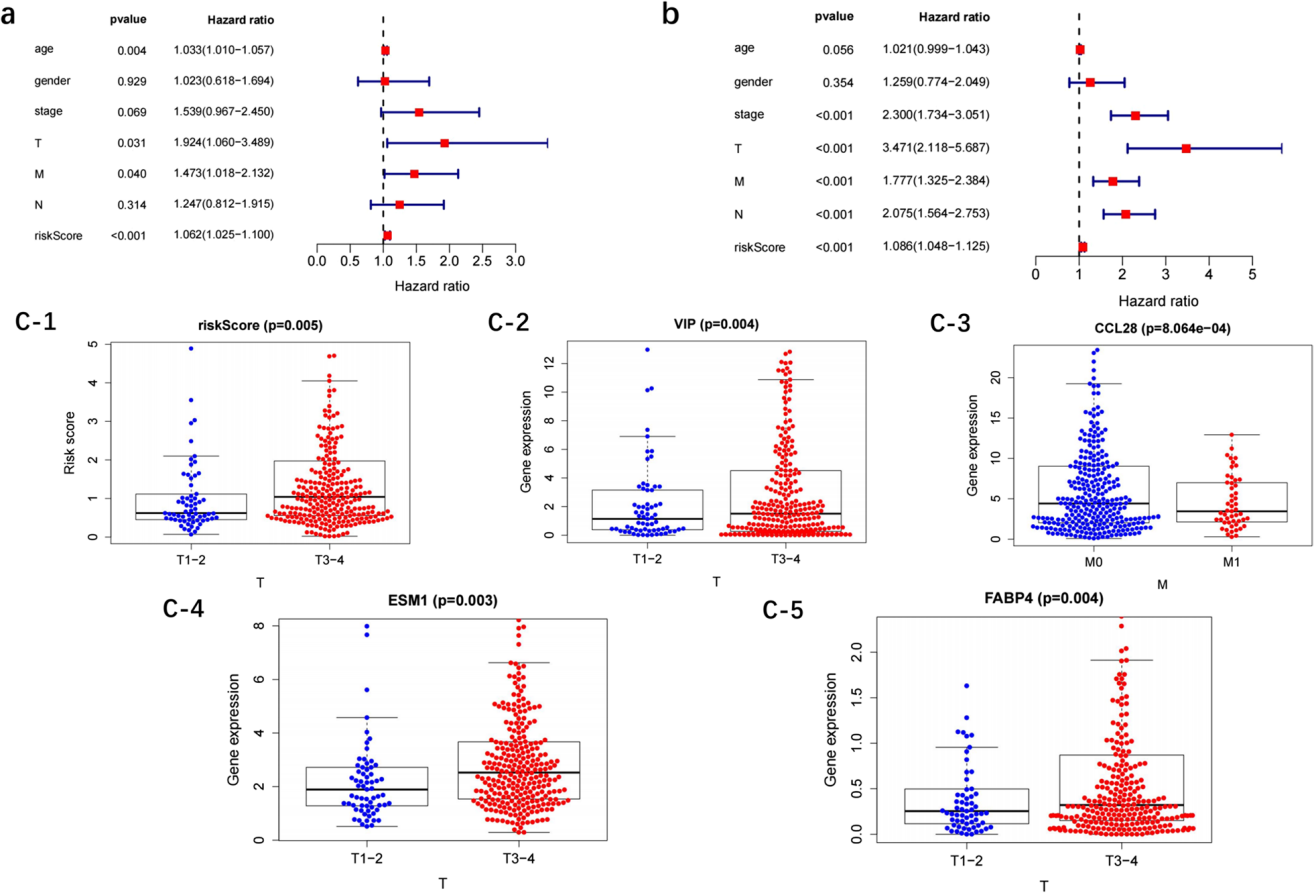
Independent prognostic value and clinical utility of the risk model. **a** Univariate analysis. **b** Multivariate Cox analyses. **c** Clinical correlation analysis of immune genes (**c-1**: Correlation analysis between the risk score and T, **c-2**: Correlation analysis between the VIP expression and T, **c-3**: Correlation analysis between the CCL28expression and M, **c-4**: Correlation analysis between the ESM1 expression and T, **c-5**: Correlation analysis between the FABP4 expression and T)

In single-factor analysis, tumor stage, T/N/M and risk score were significantly associated with prognosis (*p* < 0.05), with a HR > 1, suggesting high-risk (Fig. 6a). The multifactorial analysis of variables indicated age, T/N and risk score significantly associated with prognosis, with a HR > 1, suggesting high-risk (Fig. 6b). Besides T-stage and N-stage, risk score could be used as an independent prognostic factor to predict the prognosis of colon cancer.

For the 12 immune genes, the lower level of *CCL28* was associated with the higher M category (*p* < 0.05), whereas the higher levels of ESM1, FABP4 and VIP associated with the higher T category (*p* < 0.05) (Fig. 6c-1–c-5).

### Identification of an immune infiltration signature

**Fig. 7:**
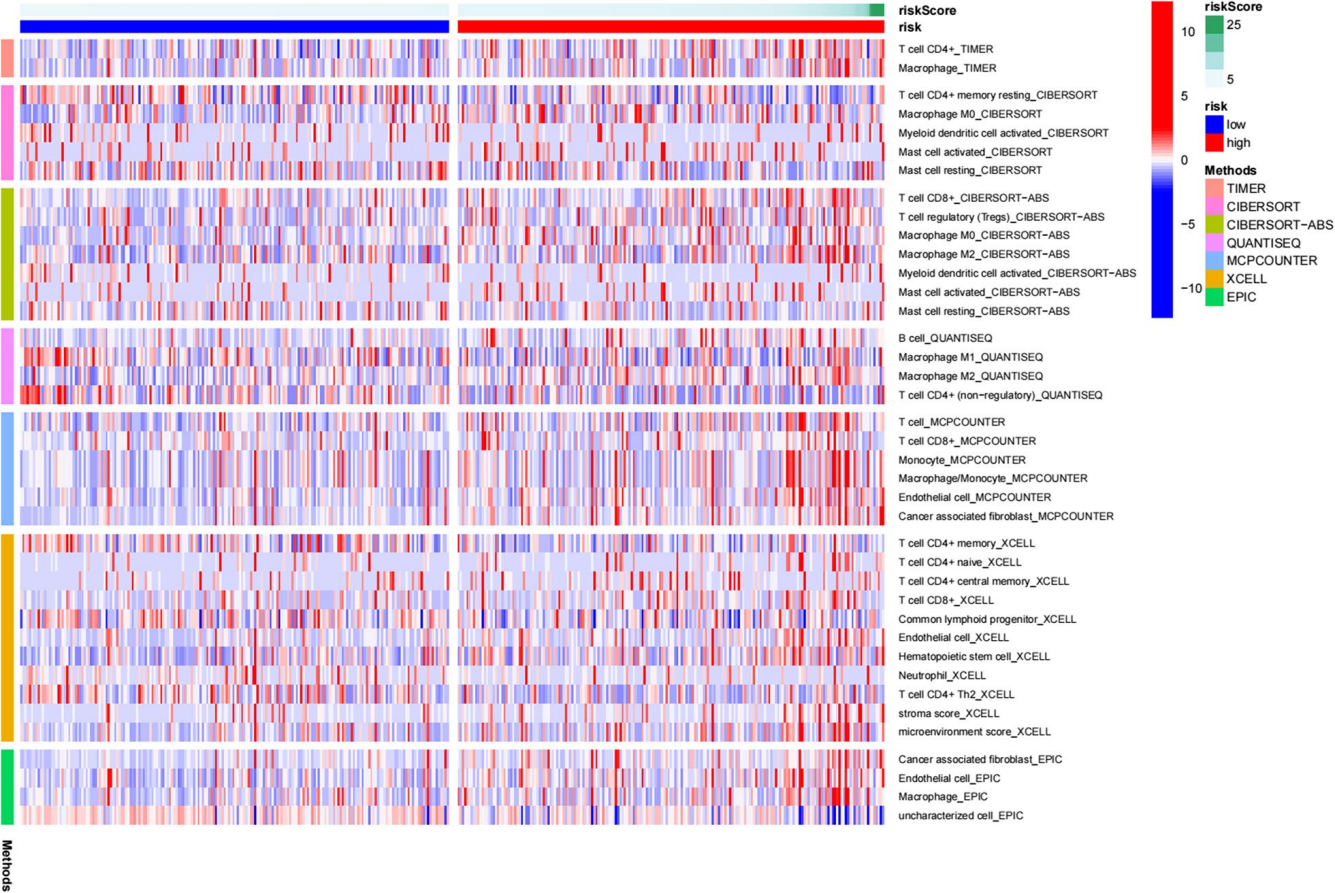
Heat map of immune cell infiltration between High and Low risk

**Fig. 8:**
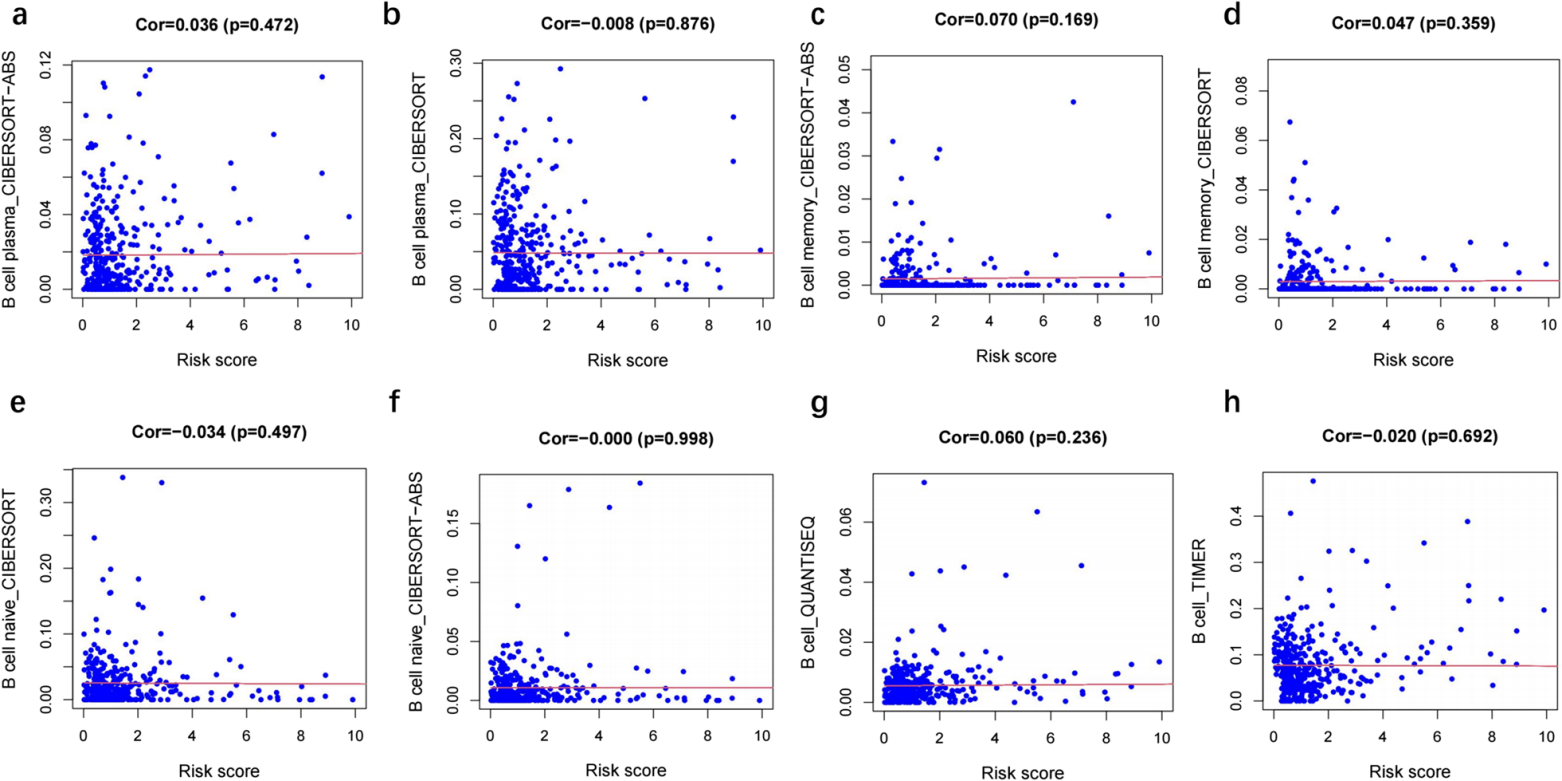
Correlation analysis between the risk score and B cell

**Fig. 9:**
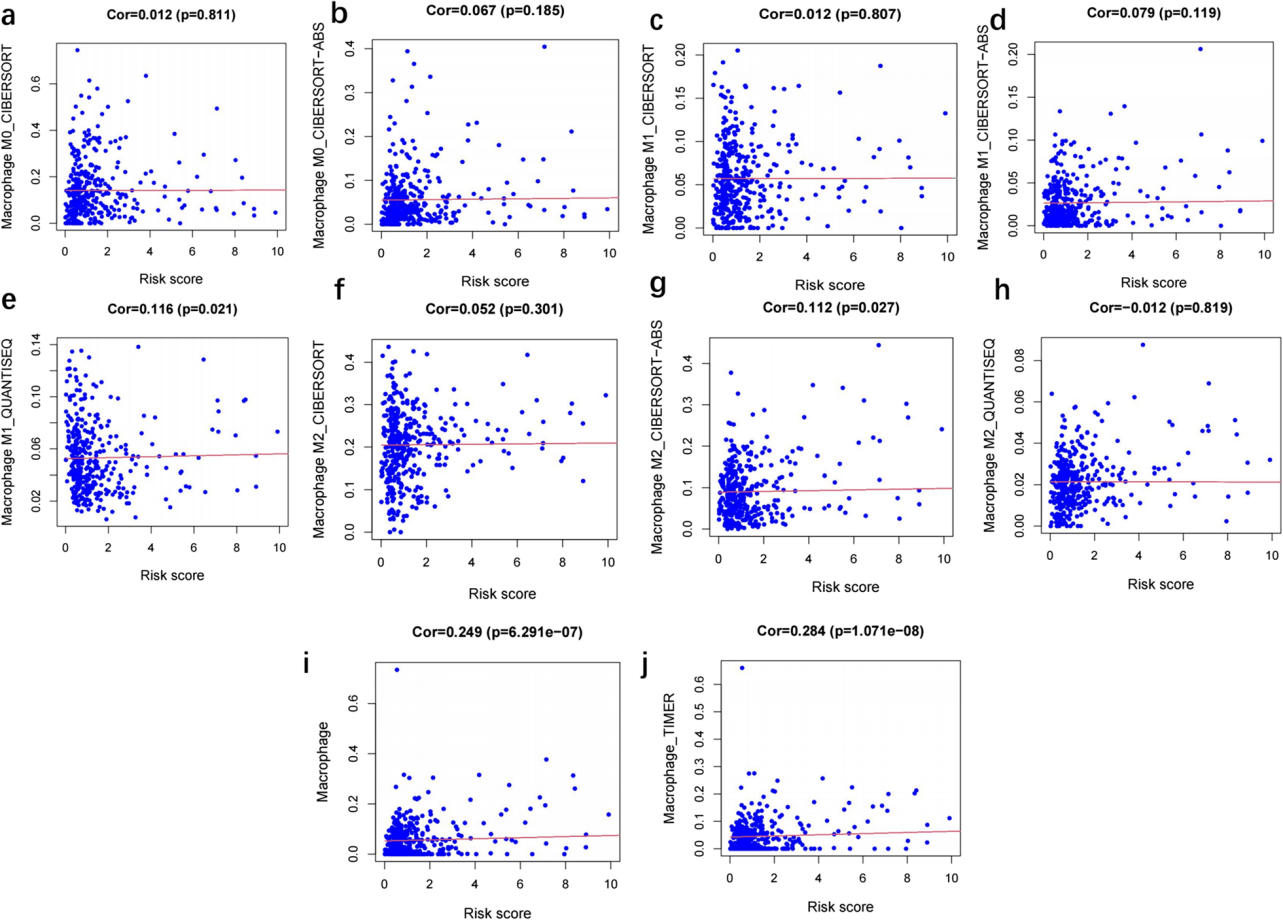
Correlation analysis between the risk score and Macrophage cell

**Fig. 10:**
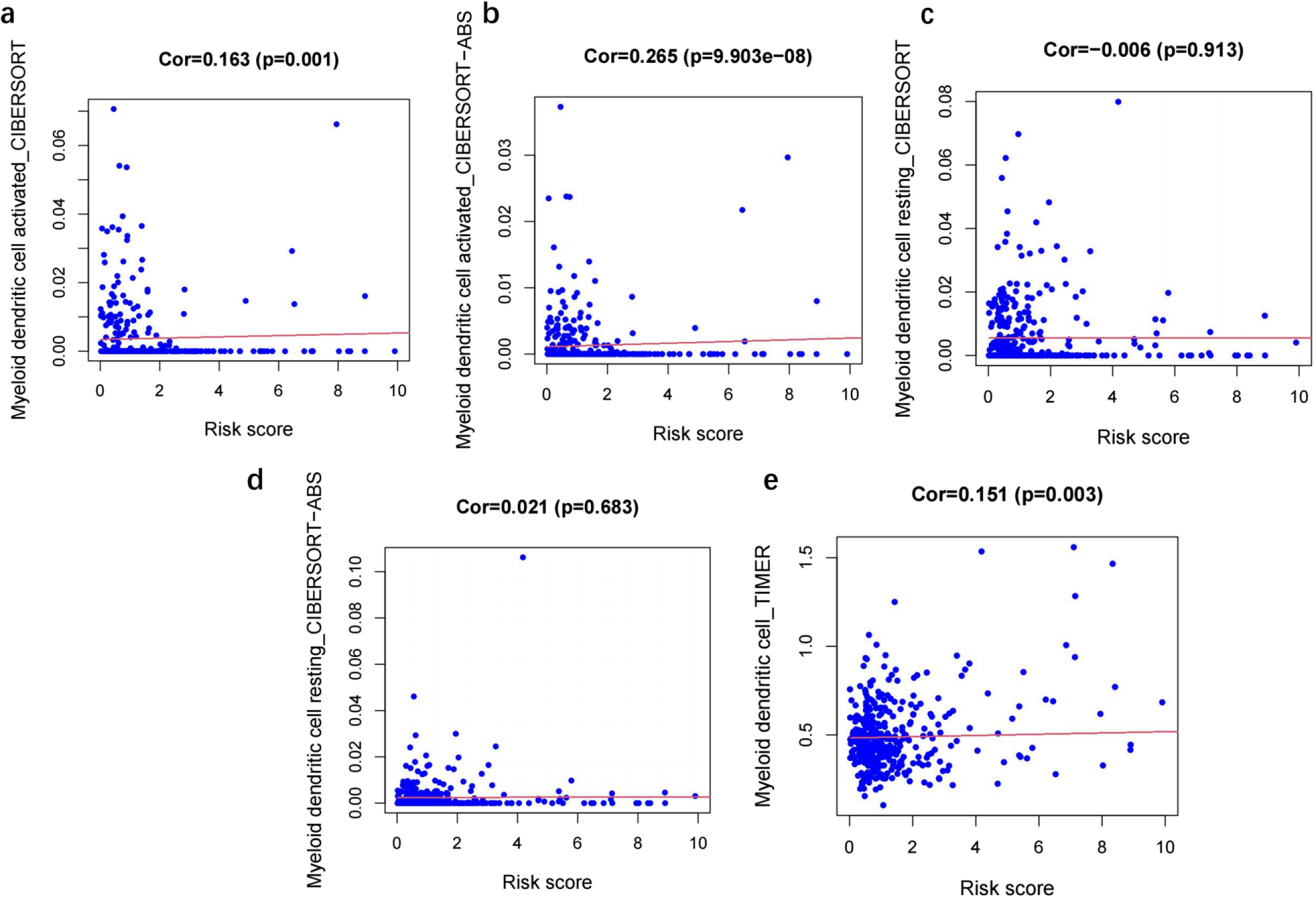
Correlation analysis between the risk score and Myeloid dendritic cell

**Fig. 11:**
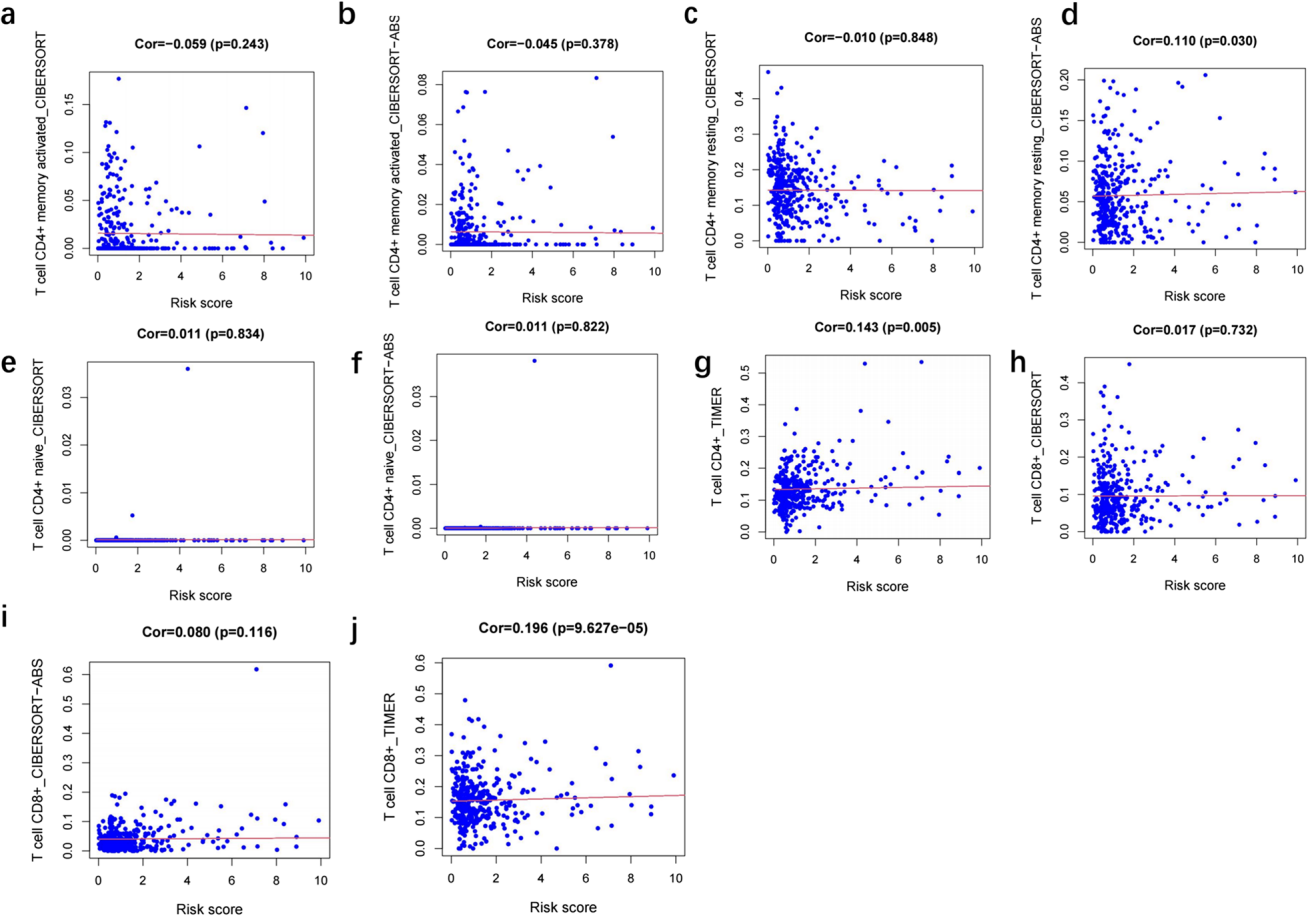
Correlation analysis between the risk score and CD4^+^T, CD8^+^T cell

Immune cell contents obtained from different databases differed slightly. The expression levels of CD4^+^ T cells, CD8^+^ T cells, neutrophils, macrophages, and dendritic cells correlated with risk scores (*p* < 0.05) (Figs. 7, 8, 9, 10 and 11), with a cor > 0, indicating that immune cell content was positively associated with a risk score. This model reflected the TME of colon cancer in which immune cells were critically involved.

**Fig. 12:**
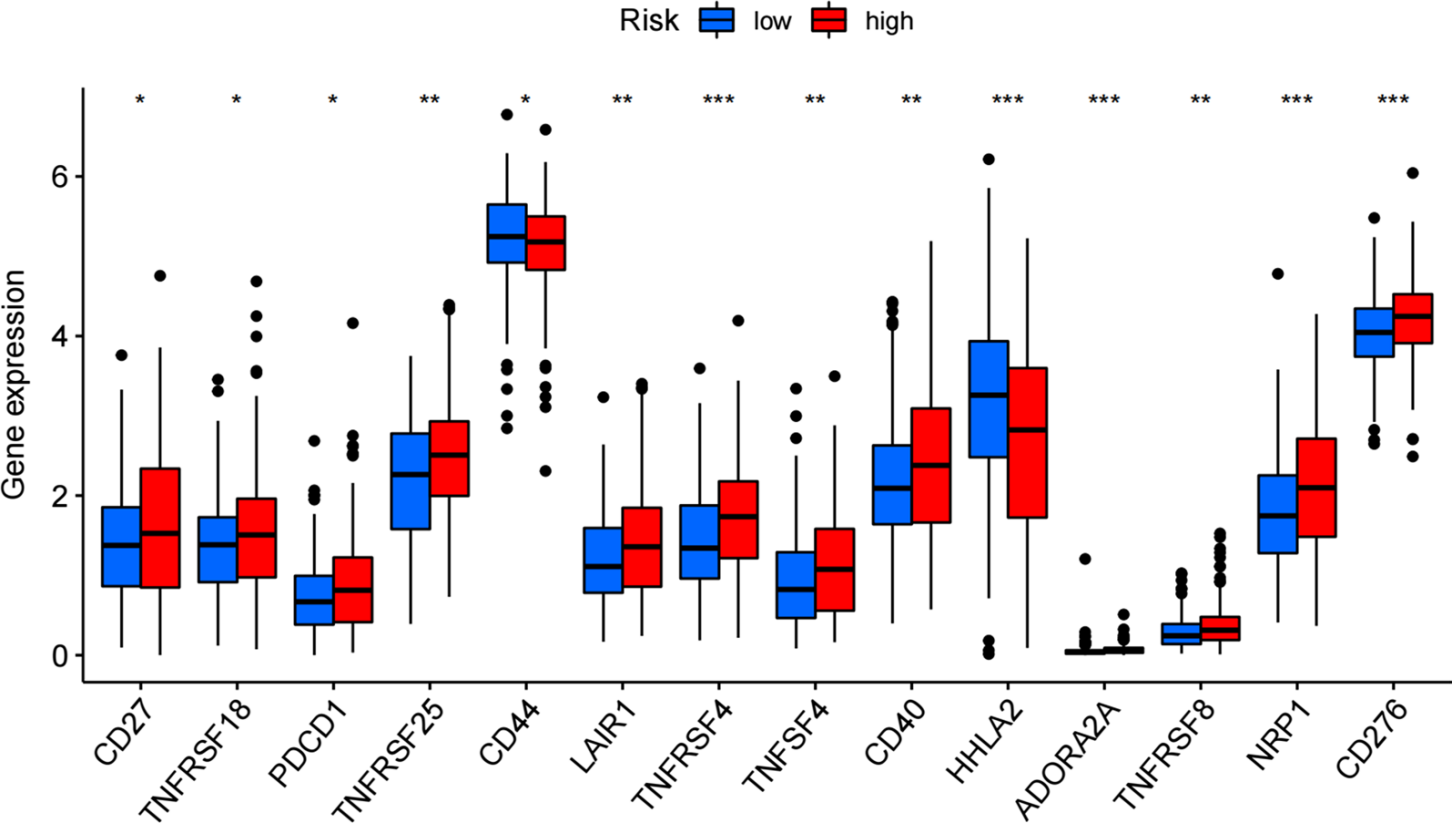
Immune checkpoint genes that differed in between high and low-risk groups

Finally, to explore new immunotherapeutic targets, we screened 14 immune checkpoint genes that differed in between high- and low-risk groups. 12 immune checkpoints were upregulated in high-risk group. CD44 and HHLA2 genes were downregulated in high-risk group. Notably, TNFRSF4, HHLA2, ADORA2A, NRP1, and CD276 were more significantly different in two groups, *p* < 0.001 (Fig. 12).

## Discussion

Colorectal cancer is currently the third most prevalent tumor worldwide, with an estimated 1.9 million new cases in 2020, accounting for 10% of all new cancer cases. Malignant tumors originating from colon predominate this population, with approximately 1.15 million new cases, 1.57 times the number of rectal cancer cases. As the second leading cause of cancer mortality, 935,000 deaths from colorectal cancer occurred worldwide in 2020, with 577,000 deaths from colon cancer, accounting for approximately 61.7% of all colorectal cancer deaths [1, 14]. The high incidence and high mortality of colon cancer have attracted more and more attention. Clinically, specific biomarkers for colon cancer diagnosis have been widely used, including serum carcinoembryonic antigen (CEA). Relevant factors affecting the prognosis of colon cancer are explored [15, 16], however, there is not yet a molecular marker for predicting the prognosis of colon cancer [17].

As a systemic disease, tumorigenesis and progression are driven by genetic and epigenetic factors, as well as complex pathways. Tumor recurrence, distant metastasis, intrinsic characteristics of tumor cells have been considered as fundamental drivers of tumor progression. With the emergence of immunotherapy, the importance of TME has been emphasized [18]. Cancer cells disrupt the integrity of intestinal barrier through interactions with immune cells, stromal cells and extracellular matrix, which together form the TME [19, 20]. The TME is not static but dynamic. The differences in drug sensitivity and prognosis reflect that colon cancer is highly heterogeneous in the TME. Generally, specific genetic and epigenetic alterations in colon cancer affect the composition of TME [19, 21, 22]. The development of colon cancer is inextricably linked to dysregulated TME. Either enlargement or reduction in cancer tissues under visual or imaging conditions are the results of interactions between cancer cells and the TME. Overall, tumor development can be divided into three stages, namely, immune surveillance in the initial stage [23], immune homeostasis in the middle stage [24] and immune escape in the final stage [25]. Tumors exhibit immune escape in the final stage as a result of an imbalance between the positive and negative forces opposing the immune system and tumor cells. Immune cells and related factors constitute important components of the TME. The type, density and location of immune cells in tumors can better predict survival [26, 27]. Tang et al. [28] classified patients into different immune subtypes according to immune components of the TME and demonstrated that immune subtypes can be used as a reliable predictor of prognosis.

In this study, we have developed an immune-related prognostic model for colon cancer and validated it as an independent predictor for prognosis. Meanwhile, TFs could be involved in prognosis-related immune gene regulation, so that a regulatory network could be constructed based on up- or down-regulation. Finally, this risk model was correlated with immune cell infiltration. Different immune cells with different expression levels presented in tumor samples. We constructed models for high- and low-risk groups. Immune functions and immune checkpoints were significantly different between high- and low-risk groups.

In this study, we have developed a prognostic prediction model for colon cancer based on 12 immune genes, including SLC10A2, FABP4, FGF2, CCL28, IGKV1-6, IGLV6-57, ESM1, UCN, UTS2, VIP, IL1RL2, and NGFR. The SLC10A2 gene encodes the apical sodium-dependent bile acid transporter (ASBT) protein, which is located in the luminal membrane of the distal ileum and proximal tubules of the kidney, and plays an important role in bile acid metabolism [29]. Downregulation of SLC10A2 could increase secretion of fecal bile acids and stimulate tumor promotion [30]. Fatty acid binding protein 4 (FABP4), a member of the intracellular lipid chaperone family, contributes to pro-tumorigenic effects of adipocytes, macrophages and endothelial cells. Adipocyte-induced FABP4 expression in ovarian cancer cells promotes metastasis and mediates resistance to carboplatin [31]. Activation of β-linked protein in gastric cancer leads to upregulation of CCL28 expression and subsequent recruitment of Treg cells thereby inhibiting gastric cancer progression [32]. Urocortins (UCNs) are members of the adrenocorticotropin-releasing factor (CRF) family, which participates in biological processes, including inflammation and cancer development [33]. Fibroblast growth factor 2 (FGF2) could promote tumor angiogenesis, migration, invasion, inflammatory response and stem cell formation in a variety of solid tumors [34]. Immunoglobulin kappa variable 1-6 (IGKV1-6) [35] and immunoglobulin lambda variable 6-57 (IGLV6-57) [36] located in the V region of the immunoglobulin light chain variable domain are involved in antigen recognition. The antigen binding sites consist of a variable region of the heavy chain and light chain, which can be somatically hypermutated. Endothelial-cells specific molecule 1 (ESM-1) [37], also known as endoglycan, is a marker of angiogenesis, involved in endothelium-dependent pathological disorders and inflammatory responses. ESM1 is overexpressed in non-small cell lung cancer, clear cell renal cell carcinoma and ovarian cancer, to regulate tumor progression. Urotensin II (UTS2) [38] regulates vasoconstriction and is associated with a range of diseases with abnormal blood pressure regulation (e.g. hypertension, kidney disease, cirrhosis, etc.). UTS2 also participates in the development of colorectal, breast, and prostate cancers. Vasoactive intestinal peptide (VIP) is a 28 amino acid peptide with a wide range of biological activities and is universally expressed in the gastrointestinal tract. VIP regulates gastrointestinal motility, modulates inflammatory responses and stimulates glandular secretion [39]. VIP behaves as a pro-metastatic factor in prostate cancer [40], whereas a protector in hepatocellular carcinoma dependent on cAMP/Bcl-xL pathway induced apoptosis [41]. Interleukin-1 receptor-like 2 (IL1RL2), also known as IL-36 receptor, is produced by monocytes and T/B lymphocytes and distributed in the intestine, kidney, skin and brain [42]. IL1RL2 plays a crucial role in inflammatory response. IL1RL2 is associated with the TEM and metastasis in breast cancer [43]. Nerve growth factor receptor (NGFR) is a member of the neurotrophin receptor family [44]. This gene induces apoptosis and is involved in injury, nervous system development and regeneration. NGFR acts as a tumor suppressor in most cancers, leading to apoptosis and suppressing metastatic invasion. However, in gliomas and melanomas, it promotes invasion and metastasis [45]. The role of NGFR in CRC requires further investigation.

To assess the predictive power of this new model, risk scores were analyzed. Overall survival time of high-risk group (with higher risk scores) was significantly shorter than low-risk group. By jointly analyzing clinical variables and risk scores, age, gender, stage, T-stage, N-stage, M-stage, and risk score were independent prognostic variables for patients with colon cancer. This model consisting of immune genes had ability to predict prognosis. Sobrero et al. [46] conducted a clinical study including 12,834 colon cancer patients and found that disease-free-survival of colon cancer patients was influenced by stage [varying from 89% (T1N1a) to T4N2b (31%)]. Another study from the Netherlands included 117,530 colon cancer patients recruited between 1995 and 2016. The 5 year relative survival rate of patients diagnosed with stage I, II and III colon cancer was 96%, 90% and 71%, respectively [47]. Patients with higher stages of colon cancer had a lower survival rate [48, 49]. Thus, survival status of colon cancer may be directly in proportional to stage. The influence of high expression of immune genes on clinicopathological factors has not yet been conclusive. By analyzing the relationship between immune infiltration and clinical traits, we observed a positive correlation between risk score and T-stage, which demonstrates the clinical applicability of this new model. Colon cancers with greater T-stage had more VIP expression. Similarly, high expression levels of the ESM1 gene and FABP4 gene were associated with T3-4. Previous studies have found a significant increase in VIP in mice with intestinal tumors through AOM/DSS induction [50]. Elevated expression of EMS1 and the chemokine CCL28 produced by intestinal mucosal epithelial cells were common in colon cancer patients. Overexpression of FABP4 promotes cell migration and invasion of colon cancer [51–54], which is consistent with our study. In this study, CCL28 gene expression was more active in M0 patients compared to colon cancer with distant metastases. At this stage, how clinicopathological factors affect the prognosis of colon cancer patients in relation to immune infiltration remains unclear. However, immune risk scores and recognized risk factors such as T-stage and N-stage constitute indispensable risk factors affecting the prognosis of colon cancer. In addition, expression levels of IGKV1-6, IGLV6-57, and CCL28 were higher in colon cancer, while VIP and FABP4 were more biased to be expressed in high-risk group. Thus, positive/negative roles of specific immune genes should be correlated with clinicopathologically relevant molecular markers.

No particular tumor can be identified by molecular markers that combine high sensitivity and high specificity. In the treatment of colon cancer, a drug targeting a specific molecule has its indications and contraindications, and there is almost no drug that can be effective for all patients. Due to heterogeneity of colon cancer, this study analyzed immune genes and downstream factors. In Figs. 7, 8, 9, 10 and 11 in this article, these cell populations were classified. By examining correlation trends of immune cells, CD8^+^ T cells and CD4^+^ T cells were positively correlated with risk score. Previous studies have suggested that immune score was used to describe the density of CD3^+^ and CD8^+^ T cell effectors in tumors and aggressive margins [26]. In contrast, Spacek et al. [55] found decreased levels of CD8^+^, CD4^+^, and NK cells and increased levels of B cells in stage II and III colorectal cancer patients by analyzing blood samples from 22 patients and 25 normal controls. CD8^+^ T cell infiltration was positively correlated with a better survival rate. Similarly, T cell memory expression was reduced, B cell memory was increased, CD4^+^ resident memory T cells and CD8^+^ resident memory T cells were reduced in colorectal cancer [56]. In this study, samples were obtained from several public databases, which was limited by the type and number of cells available. Macrophage, myeloid dendritic cell and CD4^+^ T cells positively correlated with risk scores. Macrophages promote growth of colonic malignant cells through the release of pro-inflammatory factors. Myeloid dendritic cells is essential in antitumor immunity [57, 58]. Further experiments are needed to explore the functions of immune cells.

This study has the following strengths: Firstly, a prognostic model of immune infiltration in colon cancer has been constructed by combining the prognosis of patients with immune genes using a large sample size derived from public databases. The immune risk score is an independent prognostic factor for colon cancer. The reliability of this new model has been validated by multiple methods. Secondly, associations between clinicopathological factors and immunogenes have been explored to provide more possibilities for molecular mechanism studies of colon cancer. Thirdly, important components of the TME-immune cells and TFs are extracted. This new model can predict immune cell infiltration and downstream factors expression levels. Finally, the combination of immune checkpoints and immune risk scores can provide new ideas and possibilities for immunotherapy of colon cancer patients.

There are also limitations in this study. The robustness of this immunogenetic prognostic model requires a large number of prospective clinical studies to validate our findings. The data were downloaded from public databases. It would be more convincing to supplement clinical trials to validate this model. More detailed basic experiments (both in vitro and in vivo) should be designed to support our conclusions.

## Conclusion

We developed and validated twelve immune gene models for colon cancer, including SLC10A2, FABP4, FGF2, CCL28, IGKV1-6, IGLV6-57, ESM1, UCN, UTS2, VIP, IL1RL2, NGFR. This model can be used as a tool variable to predict the prognosis of colon cancer.

## Data Availability

Several databases were used in this study, including TCGA (https://tcga-data.nci.nih.gov) database, ImmPort (https://www.immport.org/home) database, TIMER (http://cistrome.org/TIMER) database, Cistrome (http://cistrome.org/) database. Immune-cell content was downloaded from timer.cistrome.org.
